# Perceived inequality in society may not motivate increased food intake in the absence of personal socioeconomic disadvantage

**DOI:** 10.1186/s12889-023-16138-0

**Published:** 2023-06-26

**Authors:** Bobby K. Cheon, Xenia Low, Darren Jeffian Wijaya, Albert Lee

**Affiliations:** 1 Social and Behavioral Sciences Branch, Division of Intramural Population Health Research, Eunice Kennedy Shriver National Institute of Child Health and Human Development, National Institutes of Health, 6710B Rockledge Dr., Room 3166, Bethesda, MD 20817 USA; 2 School of Social Sciences (Psychology), Nanyang Technological University, Singapore, Singapore

**Keywords:** Social class, Perceived social inequality, Eating behaviors, Portion selection

## Abstract

**Background:**

Greater levels of socioeconomic inequality across societies have been associated with higher rates of obesity and cardiometabolic disease. While these relationships could be attributed to poorer quality of health services and lower access to healthier lifestyles among disadvantaged groups in societies with greater economic inequality, this explanation does not account for those who experience relative economic security in such unequal societies (e.g., the middle and upper classes). Here, we tested whether perceptions of greater disparities between social classes in one’s society (i.e., perceived societal inequality) may promote eating behaviors that risk excess energy intake.

**Methods:**

In two studies, participants completed an experimental manipulation that situated them as middle class within a hypothetical society that was presented to have either large disparities in socioeconomic resources between classes (high inequality condition) or low disparities (low inequality condition), while keeping the participants’ objective socioeconomic standing constant across conditions. In Study 1 (pre-registered), participants (*n* = 167) completed the perceived societal inequality manipulation before a computerized food portion selection task to measure desired portion sizes for a variety of foods. Study 2 (*n* = 154) involved a similar design as Study 1, but with inclusion of a neutral control condition (no awareness of class disparities) followed by ad libitum consumption of potato chips.

**Results:**

While the high inequality condition successfully elicited perceptions of one’s society as having greater socioeconomic inequalities between classes, it did not generate consistent feelings of personal socioeconomic disadvantage. Across both studies, we observed no differences between conditions in average selected portion sizes or actual energy intake.

**Conclusions:**

Taken together with prior research on the effects of subjective socioeconomic disadvantage on increased energy intake, these findings suggest that perceptions of inequality in one’s society may be insufficient to stimulate heightened energy intake in the absence of personal socioeconomic disadvantage or inadequacy.

## Introduction

Past findings have shown that lower socioeconomic status (SES) is largely associated with consumption of more energy-dense foods, higher body mass index (BMI), and obesity, albeit with some variations across societies [1–4]. One explanation is that healthier foods are often less affordable and accessible to individuals of low SES backgrounds, thus increasing consumption of cheaper and more abundant energy-dense foods [1, 2, 5]. On the other hand, recent research has demonstrated that low subjective SES (SSES), or subjective feelings of lacking socioeconomic resources relative to other people can stimulate appetite, resulting in higher food intake, even in the absence of actual SES deprivation [6, 7]. Here, we refer to appetite as the desire or drive to eat, which may be generated by subjective hunger as well as various internal or external cues (e.g., anticipated pleasure of eating, food-related cues) [8]. Although personal experiences of SES disadvantage may stimulate appetite, individuals may perceive high levels of disparities and inequalities in their broader social environment without directly feeling disadvantaged by it [9]. Thus, it remains unknown if perceptions of social inequality in one’s society are enough to stimulate appetite and increased energy intake even in the absence of personal feelings of disadvantage compared to others. The present research aims to investigate if the perception of greater levels of social inequality within one’s environment contributes to increased appetite and energy intake independent of individuals’ subjective SES.

### Subjective SES disadvantage and appetite

Prior research has identified that SSES is a strong predictor of various health-related outcomes, and these relationships are independent of objective SES indicators such as educational attainment, occupation, and actual wealth [10–12]. SSES has also been associated with health outcomes linked to appetite and dietary patterns [13, 14], which may partially explain socioeconomic disparities in outcomes such as obesity and cardiometabolic health. Recent research has also suggested that the perception of being socioeconomically disadvantaged compared to others may increase vulnerability to socioeconomic disruptions caused by the COVID-19 pandemic, which in turn may increase intentions to consume larger food portions [15].

Beyond such correlational and cross-sectional findings, research that has examined the effects of experimentally manipulated experiences of SSES disadvantage has suggested that SSES may have causal influences on appetite and eating behaviors. Notably, when participants were experimentally induced to experience disadvantage in socioeconomic standing compared to others, they consumed more calories from subsequent snacks and meals [6, 13, 16], showed greater sensitivity to energy-signaling sensory properties of beverages [17, 18], and exhibited increased circulation of the appetite-stimulating hormone ghrelin [19], even while controlling for their objective SES (e.g., income). Likewise, various experimental manipulations to generate acute feelings of personal relative deprivation (e.g., receiving unfair material outcomes compared to others) have subsequently led to the selection of larger food portion sizes and preferences for more palatable rewarding foods [7, 20].

### Perceived inequality in society, obesity, and eating behaviors

While experimental studies have demonstrated potential causal influences of perceptions of personal socioeconomic disadvantage on appetite, food preferences, and eating behavior, inequalities and disparities can also be a characteristic perceived in one’s immediate environment or society. As opposed to one’s SSES or personal relative deprivation, which captures people’s personal perceived social standing compared to others, perceived social inequality depicts the distribution of wealth across the population of a given society [9]. It encompasses differences in opportunities and outcomes to attain wealth, social standing, connections, and privileges for individuals of different social classes [21, 22]. In societies or environments with greater degrees of social inequality, differences in access to SES resources between those of higher and lower SES backgrounds may be more salient, conspicuous, and considered more meaningful.

At a societal level, prior research has demonstrated that the degree of social inequality may correlate with health outcomes across societies [23, 24]. For instance, province-level income inequality is associated with lower subjective well-being and psychological distress [25]. Likewise, obesity rates and higher BMIs across societies also associated with greater levels of income inequality [26–28].

Excess calorie intake through overeating or selection of palatable calorie-dense foods may be one behavioral mechanism in which the magnitude of inequality in society may contribute to obesity rates, even in the absence personal disadvantage or deprivation. This may be because greater levels of perceived inequality in one’s society may indirectly signal one’s environment as being harsh, resource-scarce, unpredictable, and indicating competitiveness and distrustfulness between people [9, 29, 30]. Such perceptions may motivate greater energy intake, consumption of palatable energy-dense foods, or eating in the absence of hunger [31–33]. Furthermore, such environments may also be stressful, which may contribute to stress-induced eating or selection of larger portion sizes [34–36].

Despite this prior research on the relationships between societal inequality and obesity and/or food intake, there have been limited attempts to disentangle and distinguish the effects of subjective socioeconomic standing that individuals personally experience from the effects of social inequality that individuals perceive in their societies. Although an individual is situated within a society with high levels of socioeconomic inequality (more obvious disparities in resources and opportunities between social classes), he or she may enjoy relatively high subjective socioeconomic standing within that society. Although experimental studies have suggested that perceptions of personal disadvantaged status or relative deprivation may have a causal influence on appetite and eating behaviors [7, 20], there has been limited research that sought to examine whether perceptions of greater degrees of inequality within one’s environment (i.e., societal inequality) may likewise stimulate appetite and motivations for increased energy intake. Although cross-sectional studies have shown that societies by higher degrees of income inequality may be associated with unhealthier dietary patterns and increased risk of obesity and metabolic disease [24, 26, 28], to our knowledge there have not been any experimental studies that have directly tested whether the magnitude of inequality incidentally perceived in one’s local environment may influence appetite and eating behavior.

### The present research

The present study tested whether experimentally manipulated perceptions of socioeconomic inequality in one’s environment could lead to higher levels of appetite and food intake. While structural, economic, and institutional factors may play a role in determining the dietary quality and habits of people in more unequal societies, the present research sought to isolate and test the influence of perceptions of one’s society as being more unequal. We hypothesized that when participants perceived a higher level of socioeconomic inequality in society, they would select larger food portion sizes (Study 1) and/or consume greater amounts of energy from a palatable snack (Study 2), compared to participants who perceived a lower level of socioeconomic inequality in society.

## Study 1

## Introduction

Study 1 sought to experimentally manipulate the effect of perceived inequality in one’s society on appetite. To do so, we adopted the Bimboola paradigm [37, 38]. This manipulation involved asking participants to imagine living in a new society called Bimboola, where they are provided with a middle-class income and lifestyle. We operationalized socioeconomic inequality as the differences in earnings, and material products that signal social class and privilege between the upper- and lower-income groups in Bimboola, with larger disparities between the classes representing a more unequal society. Participants’ appetite (represented by the intended amount of food intake) was then measured with a computerized Portion Selection Task in which participants selected desired portion sizes for a range of food items [7, 39]. Portion selection behavior was targeted as the outcome for Study 1 given that it represents intentions to consume greater amounts (more energy) of foods, which may reflect stronger appetite following our manipulation.

## Methods

### Participants

Using G*Power [40], at least 200 participants were required to assume a medium effect size of *d* = 0.40 and achieve 80% power (α = 0.05). A total of 204 participants were recruited from the United States through Prolific, an online platform for recruiting research participants and administering online surveys and compensated the equivalent of 3£ (pound sterling) for completing the survey. Participants who did not properly complete the survey (*n* = 16) were excluded from the study. Additionally, participants who reported currently being on a diet (*n* = 20) or did not respond to this question (*n* = 1) were also excluded from analyses, leaving a final sample of 167 participants (72 females; Age: 33.76 ± 11.63 years; BMI: 26.61 ± 7.81 kg/m^2^). The study was approved by the university’s institutional review board (IRB) and performed in accordance with the relevant guidelines and regulations. Informed consent was obtained from all participants.

### Procedure and materials

This study was pre-registered on Open Science Framework (osf.io/u6v4d) prior to completion of data collection and any analysis. Participants were told that the purpose of the study was to investigate the relationship between individual perceptions of society and food preferences before proceeding with an online survey.

### Baseline appetite

Participants first completed an appetite rating assessment by rating their levels of hunger (“How hungry do you feel right now?”), fullness (“How full do you feel right now?”), prospective food consumption (“How much do you think you can eat right now?”), and desire to eat (“How much do you want to eat right now?”), using 100-point visual analogue scales (VAS), ranging from “not at all” (0) to “very much” (100), as adapted from Flint and colleagues [41]. These items were averaged (with fullness reverse-scored) to compute a composite variable of baseline appetite (α = 0.78).

### Perceived societal inequality manipulation

**Fig. 1:**
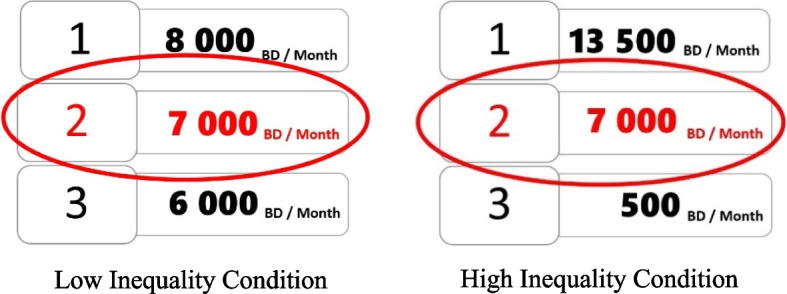
Depiction of income distributions between the upper class (#1), middle class (#2), and lower class (#3) in Bimboola in the high and low perceived societal inequality conditions of Studies 1 and 2. Participants were presented to be in the middle class (circled) in both conditions

Next, to experimentally manipulate the perception of societal socioeconomic inequality, participants were asked to imagine living in a hypothetical society called Bimboola [37, 38]. Participants were told that Bimboola was comprised of three income groups and this categorization was derived from the amount of Bimbolian Dollars (BD) that Bimbooleans earned. Participants were randomly assigned to one of two conditions: high inequality (*n* = 84) or low inequality (*n* = 83), where each condition depicted the degree of income inequality in Bimboola. In both conditions, participants saw the average monthly incomes of 3 income groups in Bimboola. High income inequality was represented by the greater income gap between the first and the third income groups in the high inequality condition compared to the low inequality condition (see Fig. 1). In each condition, participants saw the corresponding distribution of income groups and were told that they belonged to the second income group, earning 7,000 BD/month. Thus, participants’ income and social class (second income group) was identical across both the low and high inequality conditions, despite greater income disparities between the upper and lower social class groups in the high inequality condition.

To increase the saliency and reinforce the manipulation, participants were also exposed to greater (high inequality) or lower (low inequality) disparities in material and symbolic resources available to people in Bimboola. Participants were asked to select a house, a car, and a vacation destination to begin a new life in Bimboola. For each purchase category, participants were presented with images of nine options, divided into three brackets (three in each bracket) depending on their affordability to the three income groups. For example, options in the middle bracket were affordable to individuals in the first and the second income group (upper and middle class), but not to those in the third income group (lower class). Thus, participants could only select one out of six options (in the second and the third income bracket) per purchase category as they could not afford any items in the first bracket (exclusively available to the upper class). One exception was the vacation options for those in the high inequality condition. Participants could only choose one out of three vacation destinations (in the second income bracket) as there were no options in the third income bracket, representing the lower class group’s inability to afford a vacation at all under high inequality.

As in the income-related component of the manipulation, the disparities in the quality of the houses, cars, and vacation options between the high income group and low income group were much greater in the high inequality condition compared to the low inequality condition, while the options presented in the middle bracket were identical between the two conditions. For instance, in the low inequality condition, the homes available to the upper class were large and stylish single-family houses while homes available to the lower class consisted of trailers or small/modest houses. Yet in the high inequality condition, the homes available to the upper class consisted of extravagant mansions while the homes available to the lower class consisted of small houses that were dilapidated and in extreme disrepair.

This manipulation of the magnitude of perceived inequality within one’s society has been successfully applied in prior research to show that greater perceived inequality is associated with subsequent hostility to immigration [37], higher levels of independent self-construal and individualism among participants [30, 38], and perceived competitiveness that might result in higher levels of status anxiety and social vigilance [42, 43].

### Attention/manipulation check

As an attention and manipulation check, respectively, two questions were asked: “Which of the following income groups were you assigned to?” (1, 2, or 3) and “To what extent do you perceive the economic distribution in Bimboola to be unequal?” using a 100-point VAS (0 = “very equal” to 100 = “very unequal”).

### Portion selection task

**Fig. 2:**
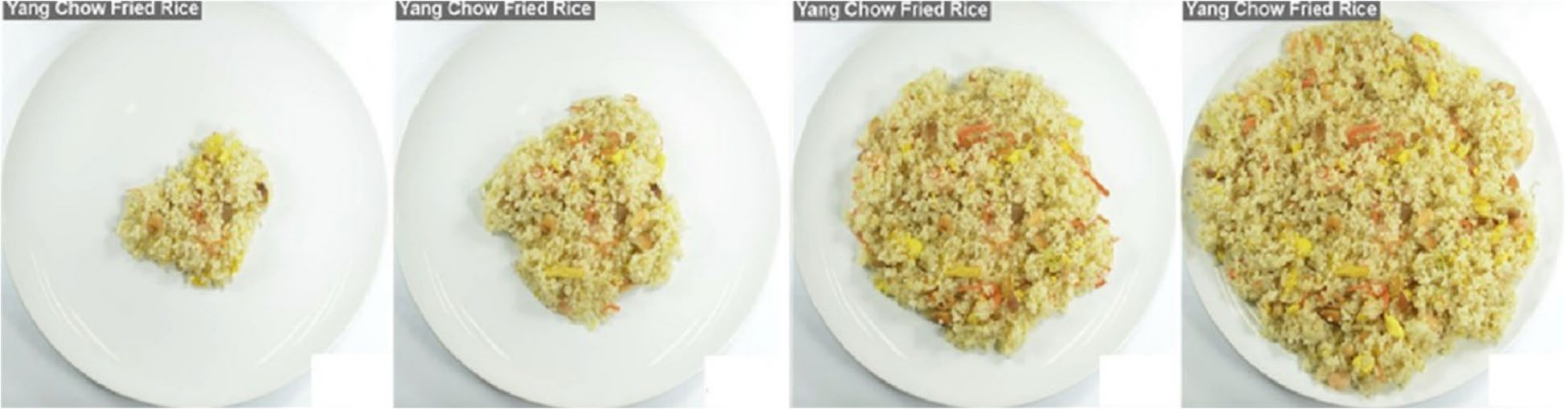
Sample food item (fried rice) and selected images from the computerized food portion selection task. For each item presented, participants could change the portion size depicted on the plate by pressing the ‘left’ and ‘right’ arrow keys. Each food item consisted of 50 portion sizes that differed by 20 kcal increments

Participants then completed an adaptation of a computerized Portion Selection Task (PST) [7, 39, 44–46]. Participants selected their ideal portion sizes of eight different food items (chicken nuggets, mixed fruit salad, mixed salad with dressing, penne pasta with tomato sauce, pepperoni pizza, Pringles original potato chips, ramen noodles, and yang chow fried rice), presented individually in a randomized order. For each food item, participants could dynamically adjust the image of the portion size displayed on the screen, with a range of 50 portion size images per item (see Fig. 2). Each successive portion size differed by 20 kcal increments, starting from 20 kcals up to 1000 kcals. Participants used a keyboard to adjust the portion sizes according to the instruction, “Your task is to select the portion of that particular food item which you would serve yourself for your next meal as a citizen of Bimboola.” The average portion size (in kcals) across the eight food items was computed.

### Subjective socioeconomic status in Bimboola

Next, participants were presented with the MacArthur Scale of Subjective Social Status [10] but reworded to refer to the society of Bimboola to assess their subjective socioeconomic standing as a citizen of Bimboola. This measure was intended to assess whether the perceived inequality manipulation was influencing societal perceptions independent of perceptions of personal socioeconomic disadvantage in Bimboola. An image of a 10-rung ladder was shown to participants with the following text:

“Think of this ladder as representing where people stand in Bimboola. At the TOP of the ladder are the people in Bimboola who are the BEST OFF — those who have the most money, most education, and most respected jobs. At the BOTTOM are the people in Bimboola who are the WORST OFF — those who have the least money, least education, and least respected jobs.

Please select the number that corresponds to the rung where you think you stand on this ladder.”

### Ratings of food items

Participants also answered how much they liked, how filling they thought, and how much they wanted to eat each of the food items presented in the PST using a 100-point VAS (0 = “not at all” to 100 = “very much”). The ratings of each of these attributes across the eight foods were computed to generate a composite variable for average liking (α = 0.63), perceived fillingness (α = 0.77), and desire to eat the food items (α = 0.63). Participants also answered how often they ate each of the items, using an 8-point Likert-type scale (1 = “never” to 8 = “once a day or more”).

### Eating behavior measures

Next, participants completed a series of measures assessing individual differences in eating behaviors. Seven items from the restrained eating subscale of the Dutch Eating Behavior Questionnaire (DEBQ) [47] were used to measure participants’ tendency to restrict food intake using a 5-point Likert-type scale (α = 0.95). Participants also completed the Tendency to Overeat Scale (TTOS) [48], which included items such as, “I stop eating when I am full” and “I know when I am full”, using a 5-point Likert-type scale (1 = “disagree” to 5 = “agree”) (α = 0.87).

### Personal relative deprivation

Participants also completed the four-item Personal Relative Deprivation Scale (PRDS) which assessed their discontent for personally feeling worse off than other people (α = 0.70) [49]. For example, participants answered “I feel resentful when I see how prosperous other people seem to be”, using a 5-point Likert-type scale (1 = “disagree” to 5 = “agree”). This measure was included to confirm that participants assigned to the low or high inequality groups did not systematically differ in chronic feelings of personal relative deprivation.

### Demographics

Participants finally stated their gender, age, height, mass, ethnicity, gross household monthly income,^1^ and whether they were dieting or having any dietary restrictions, as well as completed the MacArthur Scale of Subjective Social Status but in the context of the USA. Participants were debriefed at the conclusion of the survey.

## Results

### Participant characteristics across conditions

Overall, one-way ANOVAs and chi-squared tests (for gender) showed that participants in the high and low inequality conditions did not systematically differ in variables related to appetite, eating behaviors, or demographic background (see Table 1). No significant differences were observed between the two groups on: distribution of gender across conditions, age, BMI,^2^ baseline appetite composite, average ratings of food liking, how filling the foods were, how frequently the foods were consumed and wanting to eat the foods were that were presented in the portion selection task; DEBQ average ratings, TTOS average ratings, PRDS average ratings, and household income. The mean ratings for monthly household income of both the low and high inequality groups were in the $6000 to $7999 range. These findings suggest that participants in both conditions did not systematically differ in potentially confounding background characteristics.

**Table 1:** Study 1 participant characteristics

Variable	Low Inequality (M ± SD)	High Inequality (M ± SD)	t (or X^2^ for Gender)	df	p
Gender	*N* = 37 females *N* = 46 males	*N* = 35 females *N* = 49 males	.14	1	.704
Age	34.46 ± 11.87	33.07 ± 11.41	.77	165	.221
BMI	26.01 ± 7.77	27.21 ± 7.92	1.00	165	.160
Baseline appetite composite	42.68 ± 9.22	44.92 ± 8.21	1.66	165	.099
Liking	64.84 ± 14.80	66.59 ± 12.39	.83	165	.408
Filling	61.80 ± 13.61	61.63 ± 14.17	.08	165	.939
Frequency of consumption	3.43 ± 0.92	3.39 ± 0.88	.24	165	.811
Wanting	56.14 ± 16.08	59.70 ± 13.41	1.55	165	.123
DEBQ	2.31 ± 0.83	2.17 ± 0.71	1.17	165	.245
TTOS	3.98 ± 0.73	3.87 ± 0.86	.92	165	.357
PRDS	2.55 ± 0.87	2.70 ± 0.91	1.12	165	.263
Household income	4.75 ± 4.27	4.95 ± 4.06	.32	165	.751

*DEBQ* Dutch Eating Behavior Questionnaire, *TTOS* Tendency to Overeat Scale, *PRDS* Personal Relative Deprivation Scale

### Attention/manipulation check

All participants correctly identified that they had been assigned to the second (middle) income group in Bimboola. T-test revealed there was a significant difference between conditions on perceptions of inequality in Bimboola. Participants in high inequality condition (M = 79.42 ± 18.03) rated Bimboola as being more unequal than participants in low inequality condition (M = 54.77 ± 25.00), t(165) = 7.31, *p* < 0.001. There was, however, no significant difference between high inequality (M = 5.77 ± 1.01) and low inequality conditions (M = 5.64 ± 1.22) on perceptions of personal SES within Bimboola, t(165) = 0.78, *p* = 0.44.

### Hypothesis testing

An ANCOVA was conducted to test the effect of perceptions of inequality on the average portion size selected (in kcals) during the portion selection task while controlling for baseline appetite composite as a covariate. There was no significant difference between high inequality (M = 429.20 ± 132.92) and low inequality conditions (M = 436.75 ± 155.08) on average portion size selected, F(1, 164) = 0.27, *p* = 0.60, and thus our hypothesis was not supported.

We conducted an equivalence test [50], to determine whether the effect size for the differences in mean portion sizes selected between the low and high inequality conditions may indeed be considered statistically equivalent (rather than statistically different, as tested with an ANCOVA above). The lower and upper equivalence bounds for the effect size comparing mean portion size across the two conditions were set to d_lower_ = -0.40 and d_upper_ = 0.40, respectively. An effect size of 0.40 is the effect that the current study was powered to detect. The 90% confidence intervals of the effect size for differences in portion sizes between the low and high inequality conditions ranged from -0.31 to 0.20. Given that the confidence interval was within the lower and upper equivalence bounds, one can reject the hypothesis that the true effect is more extreme than -0.04 or 0.04, suggesting the effect of the inequality condition is statistically equivalent.

## Study 1 Discussion

Study 1 aimed to investigate the effect of perceived inequality in one’s society on appetite as measured by participants’ desired portion sizes. While the Bimboola paradigm successfully generated differences in participants’ perceptions of societal inequality, we did not observe support for our hypothesis that higher levels of perceived societal inequality would contribute to selection of larger portion sizes.

One possible explanation is that participants may not have felt personally disadvantaged even if they perceived their society (e.g., Bimboola) to be more unequal in the high inequality condition, given no significant differences in subjective SES reported within Bimboola between the two conditions. Although prior studies have found societal income inequality is associated with diet quality and obesity [26–28], these processes may emerge through more unequal environments generating feelings of personal disadvantage. This is in line with more recent observations that societal inequality may need to be perceived or experienced in order to affect health and well-being [9], possibly by contributing to impressions of personal disadvantage.

An alternate explanation for the lack of differences in portion sizes observed between conditions is that the low inequality condition may unintentionally be producing some perceptions of inequality. Although impressions of disparities between the income groups in Bimboola were much smaller in the low inequality condition (vs. the high inequality condition), the low inequality condition still exposed participants to class differences in Bimboola. Due to a lack of a neutral control condition in Study 1, we were not able to assess this possibility.

Another limitation of Study 1 was that the effect of perceived societal inequality on appetite may not be adequately captured by portion selection behavior. Although commonly consumed food items were presented in the portion selection task, there was a relatively limited selection of eight food items. It is also possible that the effects of perceived societal inequality may not necessarily manifest in planning of how much to eat, but could be expressed in other eating behaviors, such as the amount of palatable snack foods consumed ad libitum. Thus, we conducted Study 2 to address these limitations and to conceptually replicate the null findings for our hypothesis in Study 1.

## Study 2

## Introduction

Study 2 sought to confirm that perceived societal inequality does not robustly affect appetite in the absence of perceived personal disadvantage. We sought to conceptually replicate Study 1 using the same Bimboola paradigm to manipulate perceptions of socioeconomic disparities in one’s society, except with the addition of a neutral control condition (without salient class disparities). Finally, instead of measuring participants’ desired portion sizes in response to the experimental manipulation, participants’ actual food intake was measured through ad libitum consumption of a palatable, yet energy-dense snack (potato chips), given prior research suggesting that the influence of signals of scarcity or relative deprivation on eating behaviors may be more sensitive for energy-dense foods [31, 32].

## Methods

### Participants

Using G*Power [40], approximately 159 participants were required to assume a medium effect size of *f* = 0.25 and achieve 80% power (α = 0.05). Data from an initial set of participants were first collected and analysed for a student project (*n* = 79) before continuing with another round of continued data collection (*n* = 90), as analysis of the first set of data collected was required for completion of the student project. Thus, a total of 169 participants from a Singaporean university completed the survey. Participants were either compensated 5 Singaporean Dollars or awarded one research participation class credit for participation. Participants who reported being on a diet were excluded from analyses (*n* = 14). An additional participant was also excluded for completing the survey quickly with haphazard responses, leaving a final sample of 154 participants (108 females; Age: 23.16 ± 2.00; BMI: 21.23 ± 3.08 kg/m^2^). The study was approved by the university’s IRB and performed in accordance with the relevant guidelines and regulations. Informed consent was obtained from all participants.

### Procedure and materials

Like Study 1, participants were told that the purpose of the study was to investigate the relationship between individual perceptions of society and food preferences.

### Baseline appetite

Participants first completed the same baseline appetite questions presented in Study 1 (hunger, fullness, prospective food consumption, and desire to eat), using 100-point visual analogue scales (VAS), ranging from “not at all” (0) to “very much” (100) [48]. These items were averaged (with fullness reverse-scored) to compute a composite variable of baseline appetite (α = 0.92).

### Perceived societal inequality manipulation

Participants completed a similar Bimboola manipulation but were randomly assigned to one of three, instead of two, groups: high inequality (*n* = 54), low inequality (*n* = 47), or control (*n* = 53). The control condition was introduced to examine if perceived inequality generally increases appetite regardless of the magnitude of income inequality (high or low) in Bimboola. As such, the distribution of income groups in Bimboola was not shown to the control group. Rather, they began by selecting a house, a car, and a holiday destination to start living in Bimboola. Participants in the control condition were only given the three options in the middle income bracket (middle class) per purchase category, while those in other conditions (high and low inequality) could still see the options available to the low and high income groups, as in Study 1. Holiday destinations in Study 2 were also modified to fit into the Singaporean context. One example was the addition of a vacation at Genting Highlands (in nearby Malaysia) to replace the option for a motor-home road trip in the middle income bracket in Study 1. Housing and car options remained unchanged from Study 1.

### Manipulation check

Participants completed the manipulation check of perceived inequality in Bimboola used in Study 1 of the income group they were assigned to in Bimboola and the degree of economic inequality in Bimboola, although those in the control condition were not required to verify their income group.

### Subjective socioeconomic status in Bimboola

As in Study 1, participants indicated their subjective socioeconomic status within Bimboola on the MacArthur Scale of Subjective Social Status. Additionally, participants rated the extent to which they would experience 6 different feelings or emotions in Bimboola on 100-point VAS scales (0 = “strongly disagree” to 100 = “strongly agree”): “I am envious of what others have in Bimboola,” “I am grateful for what I have in Bimboola,” “What I have in Bimboola may be inadequate,” “I am happy with what I have in Bimboola,” “I feel stressed living as a citizen in Bimboola,” and “I feel anxious living as a citizen in Bimboola.” These items were included in Study 2 to assess the influence of other incidental negative or positive experiences produced by the inequality manipulation on eating behavior. The items for envy, gratefulness (reverse-scored), inadequacy, and happiness (reverse-scored) were averaged into a composite measure of the experience of emotions associated with perceived deprivation and disadvantage (α = 0.70).

### Ad libitum snack consumption

Unlike Study 1, participants were not required to complete the computerized portion selection task. Instead, they were provided with the opportunity for ad libitum snack consumption in the form of a bogus taste test [51] once they had completed the first half of the survey. Participants were presented with a bowl of approximately 75 g of potato chips (approximately 210 g total weight of chips and serving bowl combined) and instructed to consume as many potato chips as required to reliably evaluate the chips’ levels of sweetness, pleasantness, flavourfulness, blandness, and bitterness, using 100-point VAS (0 = “not at all” to 100 = “very much”). This was a bogus taste test intended to prevent potential demand characteristics from participants. Participants were also told that since any leftover chips would be disposed of after the study, they were allowed to continue eating throughout the remaining of the study. Participants then rated how much they liked, how filling they thought, and how much they wanted to generally eat chips using 100-point VAS (0 = “not at all” to 100 = “very much”). They also answered how often they ate potato chips, using an 8-point Likert-type scale (1 = “never” to 8 = “once a day or more”).

### Individual difference measures and demographics

Finally, participants completed the same items from the restrained eating subscale of the Dutch Eating Behavior Questionnaire (DEBQ) as in Study 1 (α = 0.90), Personal Relative Deprivation Scale (PRDS) (α = 0.71), and demographic questions that were presented in Study 1. The TTOS was excluded in Study 2 since it was not central to testing our hypotheses and to reduce participant burden with the amount of measures in the study. The second MacArthur Scale of Subjective Social Status was also included with respect to participants’ standing in their actual society (Singapore), rather than Bimboola. Participants were debriefed accordingly.

## Results

### Participant characteristics across conditions

Overall, participants in high inequality, low inequality, and control conditions did not systematically differ in background variables related to appetite, eating behaviors, or demographic background (see Table 2). One-way ANOVAs and chi-squared test (for gender) revealed no significant differences between the three groups on: distribution of gender across conditions, age, BMI, baseline appetite composite, average ratings of liking and how filling and wanting to eat potato chips, taste test ratings of whether chips were pleasant, sweet, bland, salty, and flavourful, DEBQ average ratings, PRDS average ratings, household income, and Singapore SSES ladder.

**Table 2:** Study 2 participant characteristics

Variable	High Inequality (M ± SD)	Low Inequality (M ± SD)	Control (M ± SD)	F (or X^2^ for Gender)	df	p
Gender	*N* = 35 females *N* = 19 males	*N* = 31 females *N* = 16 males	*N* = 42 females *N* = 11 males	3.221	2	.200
Age	20.98 ± 1.73	21.23 ± 1.96	21.28 ± 2.27	.35	2, 151	.707
BMI	20.84 ± 2.82	21.75 ± 3.59	21.17 ± 2.81	1.09	2, 150	.399
Baseline appetite composite	49.81 ± 29.16	51.30 ± 25.95	48.93 ± 26.11	.10	2, 151	.908
Liking	69.00 ± 19.34	65.38 ± 21.96	62.98 ± 20.29	1.17	2, 151	.313
Filling	48.78 ± 23.49	49.79 ± 21.37	45.94 ± 22.80	.40	2, 151	.674
Wanting	64.17 ± 20.65	58.26 ± 21.30	56.08 ± 21.96	2.06	2, 151	.131
Frequency of consumption	3.89 ± 1.27	3.13 ± 1.14	3.23 ± 1.20	6.14	2, 151	.003
Pleasant	72.22 ± 17.37	70.26 ± 18.54	69.13 ± 16.68	.43	2, 151	.653
Sweet	28.83 ± 21.34	29.38 ± 21.33	28.58 ± 21.03	.02	2, 151	.982
Bland	34.41 ± 23.46	34.19 ± 23.86	39.91 ± 20.24	1.07	2, 151	.340
Salty	57.54 ± 18.96	60.04 ± 18.93	53.87 ± 21.40	1.24	2, 151	.294
Flavourful	57.59 ± 20.05	57.26 ± 19.90	58.49 ± 18.46	.06	2, 151	.947
DEBQ	2.30 ± 0.73	2.31 ± 0.71	2.53 ± 0.85	1.17	2, 151	.312
PRDS	2.37 ± 0.82	2.35 ± 0.66	2.39 ± 0.80	.03	2, 151	.973
Household income	5.07 ± 2.89	4.68 ± 2.31	5.31 ± 2.54	.44	2, 151	.648
Singapore SSES ladder	5.89 ± 1.53	6.02 ± 1.19	6.00 ± 1.37	.14	2, 151	.871

*DEBQ* Dutch Eating Behavior Questionnaire, *TTOS* Tendency to Overeat Scale, *PRDS* Personal Relative Deprivation Scale

However, there was a significant difference between conditions on the reported frequency of consumption of potato chips, F(2, 151) = 6.14, *p* = 0.003. Bonferroni post-hoc comparisons showed that participants in the high inequality condition (M = 3.89 ± 1.27) consumed potato chips significantly more frequently than those in the low inequality condition (M = 3.13 ± 1.14), *p* = 0.006, and those in the control condition (M = 3.23 ± 1.20), *p* = 0.02. Participants in the low inequality and control conditions did not significantly differ in their frequency of consumption of potato chips, *p* = 1.000. Since the frequency of consumption was found to differ across conditions, it was included as a covariate in subsequent hypothesis tests.

### Manipulation check

Similar to Study 1, a one-way ANOVA revealed there was a significant difference between conditions on perceptions of inequality in Bimboola, F(2, 151) = 17.55, *p* < 0.001. Bonferroni post-hoc comparisons showed that participants in the high inequality condition (M = 66.54 ± 21.42) perceived Bimboola to be significantly more unequal than low inequality participants (M = 46.53 ± 21.17), *p* < 0.001, as well as participants in the control condition (M = 44.21 ± 21.34), *p* < 0.001. Participants in the low inequality and control conditions did not significantly differ in their perceptions of inequality in Bimboola, *p* = 1.00.

However, unlike Study 1, participants also significantly differed across conditions on where they placed themselves on the subjective SES ladder in Bimboola, F(2, 151) = 4.33, *p* = 0.02. Using Bonferroni post-hoc comparisons, we found that participants in the low inequality condition (M = 6.02 ± 1.05) rated their SSES in Bimboola significantly lower than those in the control condition (M = 6.66 ± 1.33), p = 0.01. High inequality participants (M = 6.28 ± 0.86) did not rate their SSES in Bimboola significantly differently than those in the low inequality condition, *p* = 0.73, or those in the control condition, *p* = 0.22.

Next, there was a significant difference between conditions on composite feelings of envy, gratefulness, inadequacy, and happiness in Bimboola, F(2, 151) = 3.42, *p* = 0.04. Using Bonferroni post-hoc comparisons, participants in the control condition (M = 32.85 ± 12.32) scored significantly lower on composite feelings of disadvantage than participants in the high inequality condition (M = 39.23 ± 15.57), *p* = 0.047. However, no difference was found between participants in the low inequality (M = 38.28 ± 12.19) and high inequality conditions, *p* = 1.00, and between participants in the low and control condition, *p* = 0.14.

Furthermore, we found a significant difference across conditions on feelings of stress in Bimboola, F(2, 151) = 4.21, *p* = 0.02. Bonferroni post-hoc comparisons revealed that participants in the low inequality condition (M = 32.60 ± 18.77) scored significantly lower than participants in the high inequality condition (M = 43.70 ± 19.67), *p* = 0.01. Participants in the control condition (M = 39.70 ± 19.46) did not score differently than high inequality participants, *p* = 0.86, and low inequality participants, *p* = 0.21.

Lastly, we found a significant difference across conditions on feelings of anxiety in Bimboola, F(2, 151) = 5.94, *p* = 0.003. According to Bonferroni post-hoc comparisons, participants in the low inequality condition (M = 28.70 ± 18.01) scored significantly lower than those in the high inequality condition (M = 41.93 ± 20.15), *p* = 0.002, but they did not score differently than those in the control condition (M = 37.23 ± 19.79), *p* = 0.09. There was no difference between the high inequality and control conditions, *p* = 0.64.

### Hypothesis testing

We tested the effect of perceptions of inequality in society on the amount of chips consumed with a one-way ANCOVA controlling for baseline appetite composite and frequency of consumption of chips as covariates. The amount of chips consumed by participants had a positive skew and was not normally distributed, as indicated by a Kolmogorov–Smirnov test of normality, *p* < 0.001. A square-root transformation was applied, which produced a normal distribution of the amount of chips consumed, *p* = 0.20. Using untransformed data, there was no significant difference between participants in the high inequality (M = 20.64 ± 18.37, low inequality (M = 18.69 ± 14.90), and control conditions (M = 19.89 ± 15.23), F(2, 149) = 0.10, *p* = 0.90. Similar results were observed using the transformed data for chips consumption: high inequality (M = 4.15 ± 1.86, low inequality (M = 3.94 ± 1.79), and control conditions (M = 4.11 ± 1.76), F(2, 149) = 0.19, *p* = 0.83.

As in Study 1, we conducted an equivalence test to determine whether the effect size for the differences in mean chips consumed between the high inequality and neutral control condition may indeed be considered statistically equivalent. We focused on these two conditions, since perceiving high inequality in society is conceptualized as a risk for increased appetite and energy intake, rather than perceiving low inequality as having an appetite-suppressing effect. As in Study 1, the lower and upper equivalence bounds for the effect size comparing outcomes across these two conditions were set to d_lower_ = -0.04 and d_upper_ = 0.04, respectively. The 90% confidence intervals of the effect size for differences in among of chips consumed between the high inequality and control conditions ranged from -0.36 to 0.27, suggesting the effect of high inequality is statistically equivalent to a neutral control condition. However, the 90% confidence interval for the effect did not fall within the equivalence bounds when comparing the effect of the low inequality and control condition (-0.25 to 0.41) or when comparing the low and high inequality conditions (-0.44 to 0.21).

Unlike Study 1, our Bimboola manipulation in Study 2 seemed to induce some feelings of personal disadvantage (e.g., placing oneself lower on the ladder of subjective SES in Bimboola, reporting higher composite feelings of disadvantage). We ran a hypothesis test while additionally controlling for subjective SES, F(2, 148) = 0.18, *p* = 0.84, and composite feelings of disadvantage, F(2, 148) = 0.13, *p* = 0.88, but still found no differences between conditions on chips consumption. Similar results were observed using the transformed data for chips consumption while controlling for subjective SES, F(2, 148) = 0.354, *p* = 0.70, and composite feelings of disadvantage, F(2, 148) = 0.24, *p* = 0.79.

## Study 2 Discussion

Study 2 was intended to verify the null results of Study 1 while addressing some limitations of Study 1. Even with the inclusion of a neutral control condition and directly measuring ad libitum snack consumption, we observed parallel results of the two studies, such that perceptions of greater levels of inequality in society did not lead to increased food intake.

Nonetheless, unlike Study 1, Study 2 generated some differences in participants’ ratings of perceived personal disadvantage or low SSES in Bimboola. Yet, these differences were largely observed between the control condition and the low inequality condition, suggesting that even lower levels of perceived disparities between social classes could generate such feelings compared to an absence of any salient socioeconomic disparities in the control condition. Finally, controlling for these feelings in our hypothesis test still revealed no unique effect of perceived inequality in society on food intake patterns, supporting the idea that disparities perceived incidentally in one’s environment may alone be insufficient to stimulate appetite.

## General discussion

The current research sought to isolate and explore the effects of perceived socioeconomic inequality in one’s society, rather than subjective personal disadvantage per se, on appetite and eating behavior. Unlike prior cross-sectional research on the relationship between society-level indicators of inequality (e.g., Gini coefficient) on aggregate diet quality or incidences of obesity across societies, the current studies were designed to test the causal effect that salient disparities between classes have on eating behavior. Both studies did not show support for the prediction that greater perceived inequality in society influences participants’ desired portion sizes and the amount of snack food consumed.

Despite the null findings, the perceived societal inequality manipulations used in the current studies were largely successful in isolating and generating impressions of one’s society (Bimboola) as being more/less unequal while minimizing influences on perceptions of personal socioeconomic disadvantage in society. While the high inequality condition consistently perceived Bimboola to be more unequal in both studies, participants in high and low inequality conditions in Study 1 did not report differences in subjective feelings of disadvantage within Bimboola. Although participants reported differences in subjective SES in Bimboola across conditions in Study 2, this experience of personal disadvantage was not consistent or confounded with perceptions of societal inequality, such that participants in the low inequality (instead of high inequality) condition reported lower subjective SES than the control condition, and the low and high inequality conditions did not differ in impressions about personal disadvantage. Furthermore, the null effect of perceived societal inequality on eating behaviors persisted even after controlling for subjective SES and composite feelings of disadvantage in Bimboola. Overall, these two studies provide a novel investigation of whether greater salience of socioeconomic disparities between classes in one’s society may stimulate appetite in the absence of perceived personal socioeconomic deprivation or disadvantage.

A notable potential implication of the null effects of perceived societal inequality is that subjective feelings of personal disadvantage may be an important component for perceived inequality in one’s society to contribute to increased appetite and energy intake. Prior cross-societal research has suggested that societies and communities marked by greater economic inequality are associated with higher incidences of obesity and poorer diets [24, 26, 27, 52], while a growing body of research using experimental methods has revealed that personal feelings of socioeconomic deprivation and unequal outcomes may have a causal contribution to motivations for increased energy intake [6, 7, 13, 16, 18, 20]. The design of the current studies falls between these two bodies of prior research, by experimentally situating individuals and their eating behaviors within a local environment perceived as more (or less) socioeconomically unequal to examine whether perceiving greater class disparities in society could contribute to similar obesogenic behaviors and dietary outcomes. The null effects along with equivalence tests in our studies may provide some reconciliation between the aforementioned bodies of literature, suggesting that salient socioeconomic inequalities may be affecting appetite and energy intake through personal feelings of disadvantage. We recommend substantiating this conclusion with further confirmatory research, such as studies that systematically examine when and how perceived inequality in society may generate personal feelings of disadvantage.

One limitation of this research is that the Bimboola manipulation and personal SES disadvantage measures were framed within a hypothetical society (Bimboola) and not within participants’ actual society. Additionally, the portion selection task was also framed to reflect portions participants would ideally consume as a citizen of Bimboola. Consequently, perceived inequality in Bimboola may be less predictive of appetite and eating behaviors in other contexts. Yet, one possible counterargument is that prior studies using the Bimboola paradigm have shown that perceived societal inequality in Bimboola may still affect participants’ real-world social judgments and attitudes [53]. Nonetheless, future research should manipulate and contextualize perceived disparities within participants’ own society (e.g., highlighting increasing inequality in one’s country) to address whether perceived societal inequalities have a unique contribution to obesogenic eating behaviors or exert their influence through some forms of personal socioeconomic disadvantage. Anxiety and stress associated with inequality, rather than perceived disadvantage produced by inequality per se, are other important considerations for future research. Finally, although we did not observe effects of perceived inequality on behaviors related to the quantity of food one may consume (desired portion sizes or amount of food consumed), perceived inequality may exert effects on other outcomes such as food preferences or choice, which may be promising targets for future research.

## Data Availability

Data can be accessed online from OSF.io. Study 1 data: https://mfr.osf.io/render?url=https://osf.io/az87f/?direct%26mode=render%26action=download%26mode=render Study 2 data: https://mfr.osf.io/render?url=https://osf.io/egvxq/?direct%26mode=render%26action=download%26mode=render

## Abbreviations

SES: Socioeconomic status
BMI: Body mass index
SSE: Subjective socioeconomic status
VAS: Visual analogue scale(s)
BD: Bimbolian Dollars
PST: Portion Selection Task
DEBQ: Dutch Eating Behavior Questionnaire
TTOS: Tendency to Overeat Scale
PRDS: Personal Relative Deprivation Scale

## Footnotes

1 Participants answered their household income on a 13-point Likert-type scale with each point indicating a range of possible income values: 1) $0-$1999; 2) $2000—$3999; 3) $4000—$5999; 4) $6000—$7999; 5) $8000—$9999; 6) $10,000—$11,999; 7) $12,000 – 13,999; 8) $14,000—$15,999; 9) $16,000—$17,999; 10) $18,000—$19,999; 11) $20,000—$21,999; 12) $22,000—$23,999; 13) more than $24,000.

2 Although BMI data were collected, a number of values were found to be too extreme and hence treated as potentially invalid (e.g., some participants may have misreported their height or weight).
